# SOX2 Promotes Radioresistance in Non-small Cell Lung Cancer by Regulating Tumor Cells Dedifferentiation

**DOI:** 10.7150/ijms.75315

**Published:** 2023-04-29

**Authors:** Shennan Wang, Zhou Li, Piao Li, Lingling Li, Yu Liu, Yanqi Feng, Ruichao Li, Shu Xia

**Affiliations:** 1 Department of Oncology, Tongji Hospital, Tongji Medical College of Huazhong University of Science and Technology, Wuhan, China; 2 Xiangyang Central Hospital, Xiangyang, China

**Keywords:** Non-small cell lung cancer, radioresistance, cancer stem cell, SOX2

## Abstract

**Background:** Radiation therapy plays an important role in the treatment of patients with non-small cell lung cancer (NSCLC). However, the radiocurability is greatly limited because of radioresistance which leads to treatment failure, tumor recurrence, and metastasis. Cancer stem cell (CSC) has been identified as the main factor that contributes to radiation resistance. SOX2, one of the transcription factors specifically expressed in CSC, is involved in tumorigenesis, progression, and maintenance of cell stemness. But the association between SOX2 and NSCLC radioresistance is not clear now.

**Methods:** We constructed the radiotherapy-resistant cell line of NSCLC by multiple radiotherapy treatments. Colony formation assay, western blot, and immunofluorescence were performed to detect the radiosensitivity of cells. Western blot, qRT-PCR, and sphere formation assay were used to detect CSC characteristics of cells. Wound healing assay and Transwell assay were used to determine cell migration motility. The SOX2-upregulated model and SOX2-downregulated model was constructed by lentivirus transduction. Finally, the expression and clinical relevance of SOX2 in NSCLC were investigated by bioinformatics analysis based on TCGA and GEO datasets.

**Results:** The expression of SOX2 was increased in radioresistant cells and a trend of dedifferentiation were observed. The results of wound healing assay and Transwell assay showed that SOX2 overexpression significantly promote the migration and invasion of NSCLC cells. Mechanistically, overexpression of SOX2 enhanced radioresistance and DNA damage repair capability of parental cells, while down-regulation of SOX2 led to decreased radioresistance and DNA repair ability in radioresistant cells, all of which were related to cells dedifferentiation regulated by SOX2. In addition, bioinformatics analysis show that high expression of SOX2 was strongly associated with the progression and poor prognosis of patients with NSCLC.

**Conclusions**: Our study revealed that SOX2 regulates radiotherapy resistance in NSCLC via promoting cell dedifferentiation. Therefore, SOX2 may be a promising therapeutic target for overcoming radioresistance in NSCLC, providing a new perspective to improve the curative effect.

## Background

Lung cancer has a very high incidence and mortality rate and is one of the most threatening malignancies to human health worldwide. More than 85% of cases are classified as non-small cell lung cancer (NSCLC). NSCLC can be further classified into three histological subtypes: lung adenocarcinoma (LADC), squamous cell carcinoma (LSCC), and large-cell carcinoma (LCC) 1, 2. With the continuous improvement of NSCLC treatment modalities, there are various treatment directions, such as surgery, chemotherapy, radiotherapy, immunotherapy, and biologically targeted therapy 2. For patients with advanced NSCLC or otherwise unable to undergo surgery, radiotherapy remains one of the most important treatments, mainly through ionizing radiation that causes DNA double-strand breaks and thus lethal cells 3. In clinical, fractionated radiation is the most common treatment modality; however, some patients with NSCLC show varying degrees of radioresistance, which greatly limits their therapeutic efficacy and leads to local tumor recurrence or distant metastasis 4. It is commonly believed that the radioresistance of tumor cells is associated with tumor heterogeneity 5, but its exact mechanism remains unclear. Therefore, it is particularly important to further explore the mechanism of NSCLC radioresistance for clinical treatment and patient prognosis.

Recently, it has been found that there is a heterogeneous class of cells with stem cell characteristics in tumors - cancer stem cells (CSC), which are undifferentiated cells with unlimited self-renewal ability and high tumorigenicity. CSCs account for a very small amount of tumor tissue but play an important role in tumor growth, recurrence, and metastasis 6. CSCs were first identified in leukemia, and researchers found that only implantation of CD34 + CD38-acute leukemia (AML) cells into immunodeficient mice triggered leukemia 7). CSC has been identified in a variety of malignancies including breast cancer 8, head and neck tumors 9, gastric cancer 10, prostate cancer 11, pancreatic cancer 12, colon cancer 13, and lung cancer 14. CSC is considered to be a highly treatment-resistant class of cell subpopulation and a major factor causing radiotherapy failure 15. CSC has been found to exhibit greater radiation tolerance in glioma, breast, cervical, and prostate cancers 16-18. It has been reported that the radiotherapy sensitivity of tumor cells can be influenced by the cell proliferation cycle, i.e., cells in the division phase (M phase) are more sensitive to radiation, while CSC is mostly in the relatively quiescent G_0_ phase, thus enhancing their radiation resistance 19. Reactive oxygen species (ROS) is a key mediator in the process of radiotherapy-induced cell damage 20, and the hypoxic state of CSC can reduce the intracellular ROS level and enhance the tolerance of CSC to radiation 21. After radiotherapy CSC can rapidly repair DNA damage through ATM and Chk1/Chk2 phosphorylation and activate WNT, Notch, and other signaling pathways to reduce apoptosis 22. In addition, CSC can be regulated by the microenvironment. The tumor microenvironment can maintain cell stemness to protect CSC from radiation damage, further promoting CSC resistance to radiotherapy 23. Common CSC-specific surface markers include CD24, CD44, CD133, and ALDH 24. Among them, CD133 is a transmembrane glycoprotein that was originally used to specifically label hematopoietic stem cells 25. ALDH is a detoxifying enzyme that is involved in intracellular metabolic processes. These two molecules have been identified as biomarkers of CSC in lung cancer 14, and high expression of these markers is usually associated with poor prognosis after radiotherapy in lung cancer patients 26. The transcription factor SOX2 (sex-determining region of Y chromosome related high mobility group box2, SOX2) is a member of the SOX gene family and has now been identified as a key factor required for the regulation of cell growth and differentiation in developing mouse embryos 27. SOX2 is also a major factor in maintaining stemness in embryonic and adult stem cells and is extremely important for tumor development as well as maintaining the undifferentiated state of cancer cells and is considered as one of the stemness genes specifically expressed in CSC 28. Takahashi et al. found that SOX2 induces reprogramming of human fibroblasts together with other stemness-related transcription factors 29. In skin and colorectal cancers, SOX2-expressing tumor cells were found to exhibit more pronounced stemness characteristics and high tumorigenicity 30. A large enrichment of SOX2 was found in osteosarcoma stem cells, and by reducing SOX2 expression tumor cells can lose their tumorigenicity and regain their ability to differentiate cells 28. High expression of SOX2 was observed in lung cancer in association with tumor metastasis, and inhibition of SOX2 expression hindered tumor cell self-renewal 31. In addition, radiotherapy was found to affect the expression level of SOX2. In hepatocellular carcinoma, radiation-induced elevated SOX2 expression in tumor cells 32. In NSCLC, SOX2 expression was significantly increased in cells after radiotherapy and showed stemness characteristics 33, 34. Further studies found that SOX2 expression was higher in tumor cells with relatively low sensitivity to radiotherapy, for example, in prostate cancer, SOX2 expression was upregulated in tumor cells that survived fractionated radiotherapy 35. In cervical cancer, SOX2 expression was significantly increased in radiation-resistant cells screened by multiple radiotherapy treatments 36. However, the relationship between SOX2 and radiotherapy resistance in NSCLC and the specific regulatory mechanisms remain unclear.

## Materials and methods

### Cell culture and treatment

Human H460 NSCLC cells (CVCL_0459) were obtained from the Laboratory of Department of Oncology, Tongji Hospital, Huazhong University of Science and Technology (Wuhan, China). They were cultured in Roswell Park Memorial Institute (RPMI)-1640 medium (HyClone, ThermoFisher) containing 10% fetal bovine serum (FBS, Gibco, ThermoFisher) at 37°C with a humidified 5% CO2 environment.

### X-ray irradiation

Cell irradiation was performed using an X-ray irradiator (RS 2000 Biological System irradiator, 25 mA, 160 kV; Rad Source Technologies Inc.) by administering a single 6Gy dose of radiation. The cells were exposed to single irradiation of 6Gy once a week, 35*35cm irradiation field, 100cm source skin distance, 1cm compensator, and a rack angle of 180°. After radiotherapy, cell culture dishes were placed in an incubator to continue the culture until the next radiotherapy. The H460 parent cells (H460-PT) were treated with a total of 72 Gy ionizing radiation and considered as radioresistant cells (H460-RR).

### Colony formation assay

Cells were seeded at 100, 500, 1000, 5000, and 10000 per well in 6-well plates. The plates were incubated at 37 °C overnight for attachment and then irradiated with 0, 2, 4, 6, 8 Gy. After irradiation, the six-well plates were placed in a cell culture incubator for 2 weeks. Then cells were fixed with methanol and stained with 0.1% crystal violet. The plates were photographed, and for clone counting, the number of cells above 50 was counted as one clone, and the colony formation efficiency (PE) and survival fraction (SF) were calculated. Survival curves of the cells were plotted by a single-hit multi-targeted model (y = 1-(1-exp(-k*x))ˆN). The sensitizing enhancement ratio (SER) was used to evaluate the radiation sensitizing effect.

### Western blot

Cell total protein was extracted by RIPA buffer and the concentration was determined by BCA reagent kits. Then proteins were separated by SDS-PAGE and transferred to polyvinylidene difluoride membranes. Blots were probed with primary antibodies overnight. After secondary antibodies incubation, each blot was captured by SynGene G: Box Chemi XT4.

### Real-time PCR

Total RNA was extracted from cells using Trizol reagent (TAKARA) and then was reverse-transcribed into cDNA. Real-time (RT) PCR was performed by RT-PCR reagent kit (TAKARA). And the results were calculated using the formulation 2^-△△Ct^. The primers were designed in PrimerBank as follows:

SOX2: Forward (5'-3')-GGATAAGTACACGCTGCCCG, Reverse (3'-5')-ATGTGCGCGTAACTGTCCAT.

CD133: Forward (5'-3')-TCCATGGCAACAGCGATCAA, Reverse (3'-5')-ATTGAGAGATGACCGCAGGC.

ALDH: Forward (5'-3')-GCACGCCAGACTTACCTGTC, Reverse (3'-5')-CCTCCTCAGTTGCAGGATTAAAG.

GAPDH: Forward (5'-3')-GCTGAGTACGTCGTGGAGTC, Reverse (3'-5')-GGGCAGAGATGATGACCCTT.

### Sphere formation assay

Cells were plated in 24-well ultralow attachment plates (Corning, Corning, NY) at a density gradient of 1000, 2000, 4000 cells per well with the medium containing 20% BIT9500 serum, 20 ng/mL epidermal growth factor, and 20 ng/mL recombinant basic fibroblast growth factor. The numbers of spheres with a diameter of 100 um or more, were observed and calculated by 3 different fields of view under the microscope after 7 to 10 days.

### Wound healing assay

Cells (5*10^5^) were placed in 6-well plates with 10% fetal bovine serum and grown to a monolayer overnight. Then the 10 ul pipette tip was used to draw a straight line on the bottom of 6-wells plates. And the cells were incubated in RPMI-1640 medium with 2% serum. Cells' shifting distances were photographed under microscopy at 0 h, 24 h, and 48 h.

### Transwell assay

Transwell migration assays were performed through 24-well plates with an 8-um diameter chamber (Corning, Corning, NY). Cells (5*10^4^) were seeded in the upper chamber with 200ul serum-free RPMI-1640 medium. And added 600ul of medium containing 20% FBS to the lower chamber and incubated for 24h. Cells on the underside of the chamber were observed and counted under microscope (three fields per chamber).

### Immunofluorescence assay

Cells were seeded in 24-well plates. After irradiation, cells were washed and fixed with 4% paraformaldehyde. And add 100ul Triton to each well for membrane breaking. Then 5% BSA was added to each well to cover the cells for 30 min. Cells were subsequently incubated with diluted primary antibodies overnight at 4℃. And cells were then incubated with secondary antibodies. After nuclear staining with DAPI, cells were viewed under the fluorescence microscope.

### Cell transduction

The lentiviral vector containing SOX2 sequence, empty vectors, SOX2 shRNA, and control shRNA were purchased from OBIO (Shanghai, China). H460-PT and H460-RR cells were used for transduction following the instructions. Cells were then cultured in RPMI-1640 medium with 2 mg/mL puromycin to select stable cell lines.

### Publicly-available databases and bioinformatic analysis

The gene expression and clinical information of NSCLC samples were downloaded from The Cancer Genome Atlas (TCGA) database and Gene Expression Omnibus (GEO). The TCGA dataset (n=703) were used to analyze the SOX2 expression in NSCLC and normal lung tissues. Samples in two GEO datasets (GSE30219 n=293; GSE37745 n=194) were divided into the low SOX2 group and high SOX2 group according to the median level of expression. To investigate the SOX2-related biological functions, we further performed Gene Ontology (GO) analysis of SOX2 co-expression genes through the cBioportal database and DAVID database. The study was conducted in accordance with the Declaration of Helsinki (as revised in 2013).

### Statistical analysis

Graphpad Prism 8.0 was used for graph production and data analysis. All results were presented as means of at least 3 independent experiments and experimental data were expressed as mean ± standard deviation (x ± s). Student's t-test was used to analyze the differences between the two groups. The OS of NSCLC patients with different expression levels of SOX2 was determined by Kaplan-Meier analysis. P < 0.05 was considered statistically significant.

## Results

### Establishment and verification of NSCLC radioresistant cell line

**Figure 1:**
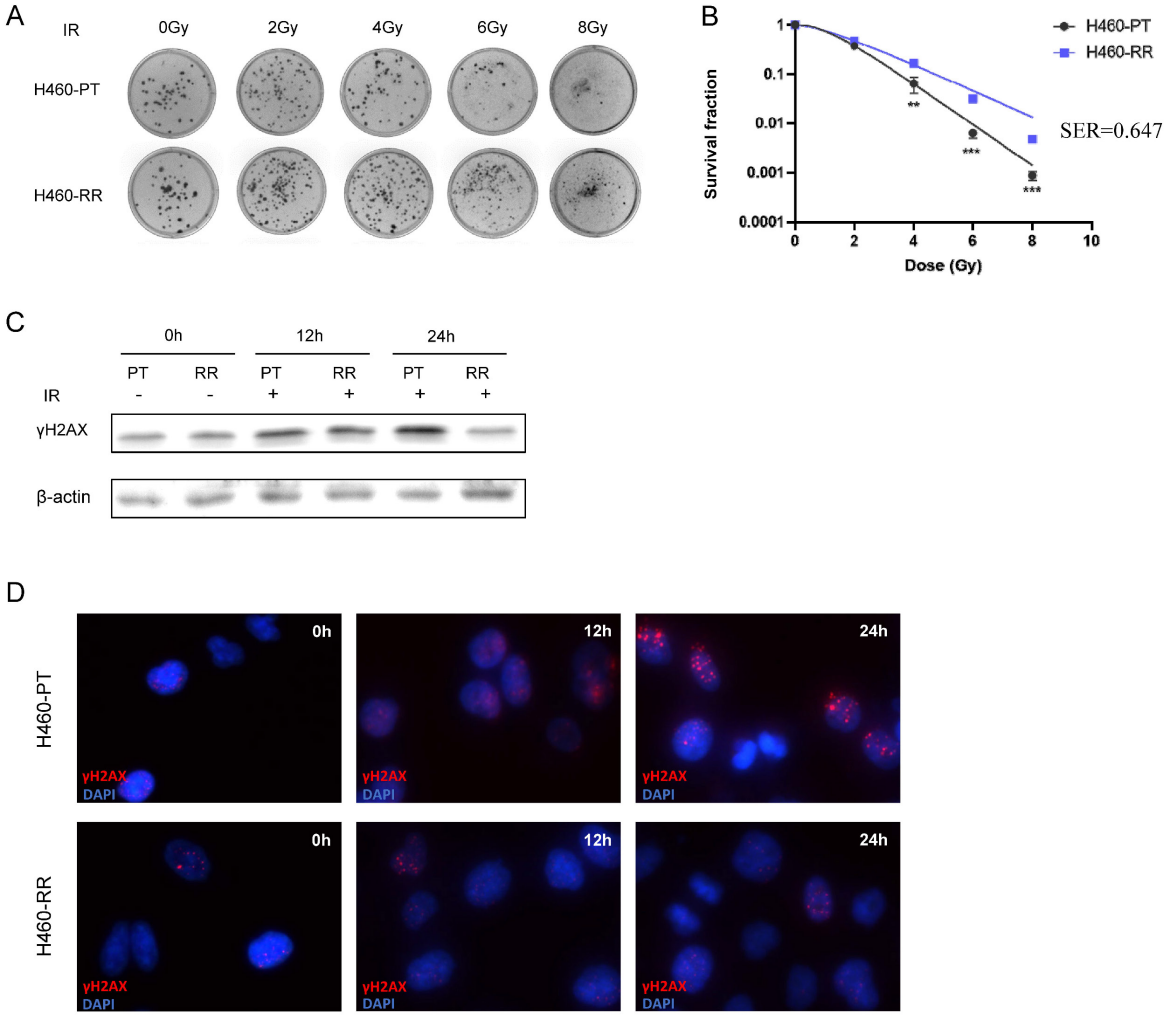
** Verification of NSCLC radioresistant cell line.** (**A**) Colony formation assay was performed to detect cell clone formation ability of H460-PT and H460-RR after radiation. (**B**) Survival fraction between H460-PT and the H460-RR was calculated. (**C**) γH2AX expression level of H460-PT and H460-RR after irradiation with 6Gy was detected by western blot. (**D**) γH2AX expression level of H460-PT and H460-RR after irradiation with 6Gy was detected by immunofluorescence.

To explore the characteristics and mechanism of radioresistance in NSCLC, the H460 cell line was selected to establish the radiation-resistant cell model. The H460 parent cells (H460-PT) were treated with a total of 72 Gy ionizing radiation and considered as radioresistant cells (H460-RR). The radiosensitivity of H460 was firstly measured by colony formation assay. Compared with H460-PT, H460-RR showed more cell colonies after radiation (**Figure 1A**). And the survival fraction of H460-RR was higher than that of H460-PT (p<0.05; **Figure 1B**). Sensitizing enhancement ratio (SER) refers to the ratio of a single radiation dose required to achieve a specific biological effect with irradiation combined with a radiation sensitizer to achieve the same biological effect. SER was used to evaluate the radiation sensitizing effect.

The presence of phosphorylated histone H2AX (γH2AX) is a crucial marker of DNA double-strand break, and the expression of γH2AX is positively correlated with DNA damage induced by radiotherapy 37. Western blot was performed to detect the γH2AX expression level in H460-PT and H460-RR after radiation. The results indicated that the expression level of γH2AX increased in both groups after radiotherapy. However, the γH2AX expression in H460-RR was lower than that in H460-PT 12h later. And after 24h, the γH2AX expression level of H460-RR was almost restored (**Figure 1C**). Moreover, a similar result was also verified in the immunofluorescence assay (**Figure 1D**), which elucidated that H460-RR possessed higher DNA repair capacity. Therefore, H460 cells exposed to a total of 72gy irradiation showed higher survival fraction and DNA repairability, which were our expected radioresistant cells H460-RR.

### The migration of H460-RR was enhanced

**Figure 2:**
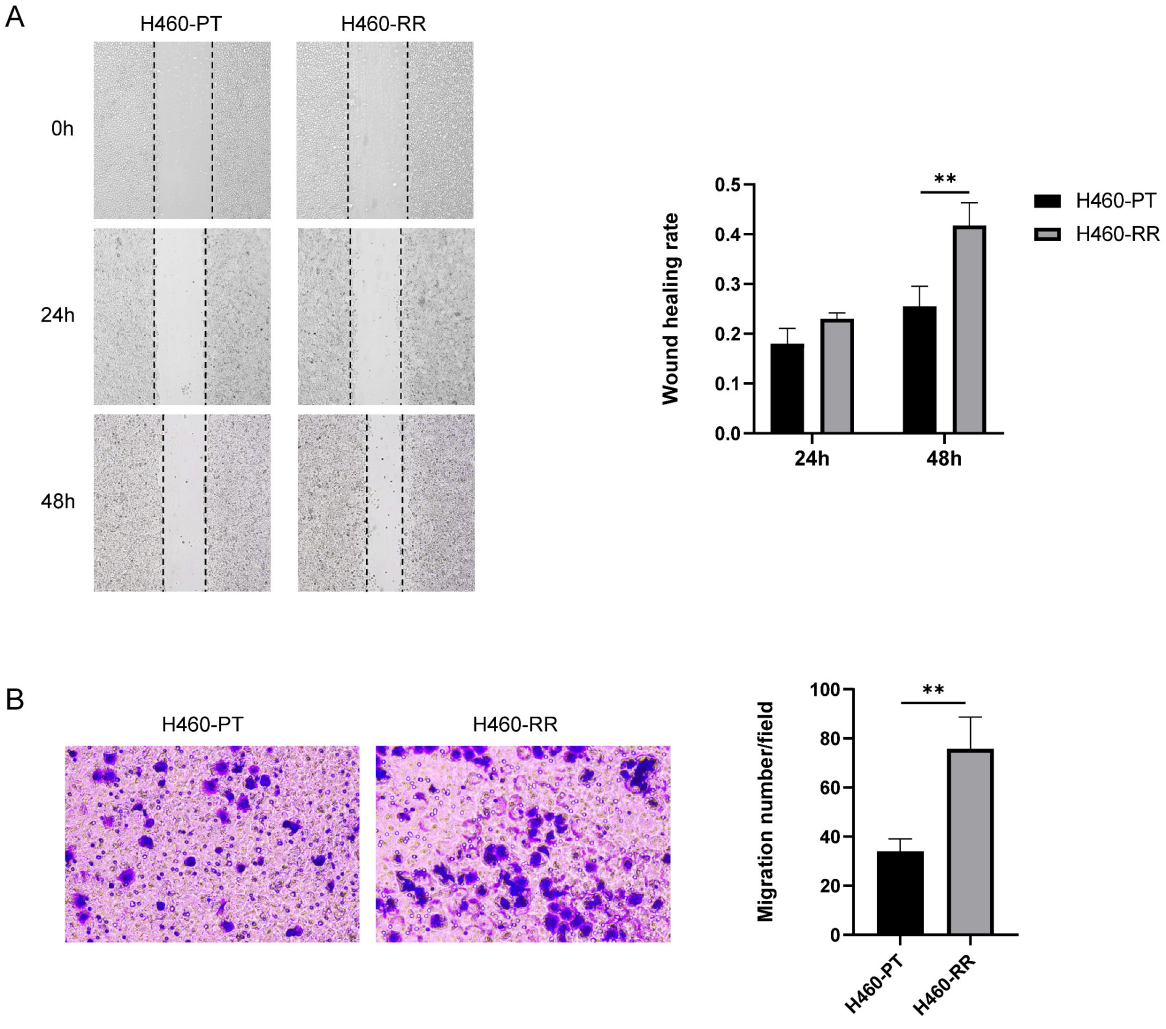
** The migration of H460-RR was enhanced.** (**A**) wound healing assay to detect the migration ability of H460-PT and H460-RR at different times after scratching. (**B**) Transwell assay to detect the migration ability of H460-PT and H460-RR.

To compare the migration ability of H460-PT cells and H460-RR cells, a wound-healing assay was performed on the two groups of cells. And the migration distances of the cells were observed under the microscope and photographed sequentially at 0, 24, and 48 h after scratching to calculate the healing percentage of the cells. The results showed that the scratch healing rate of H460-RR was higher than that of H460-PT, and the difference between the two groups was especially significant at 48h (p<0.05, **Figure 2A**). The migration ability of the cells in both groups was verified again by Transwell, and the results showed that the number of cells crossing the chambers in H460-PT was significantly less than that in H460-RR (**Figure 2B**), demonstrating that the migration motility of the cells in the radioresistant group was higher than that of the parental cells.

### Characterization of CSC phenotype was increased in RR-H460 cells

**Figure 3:**
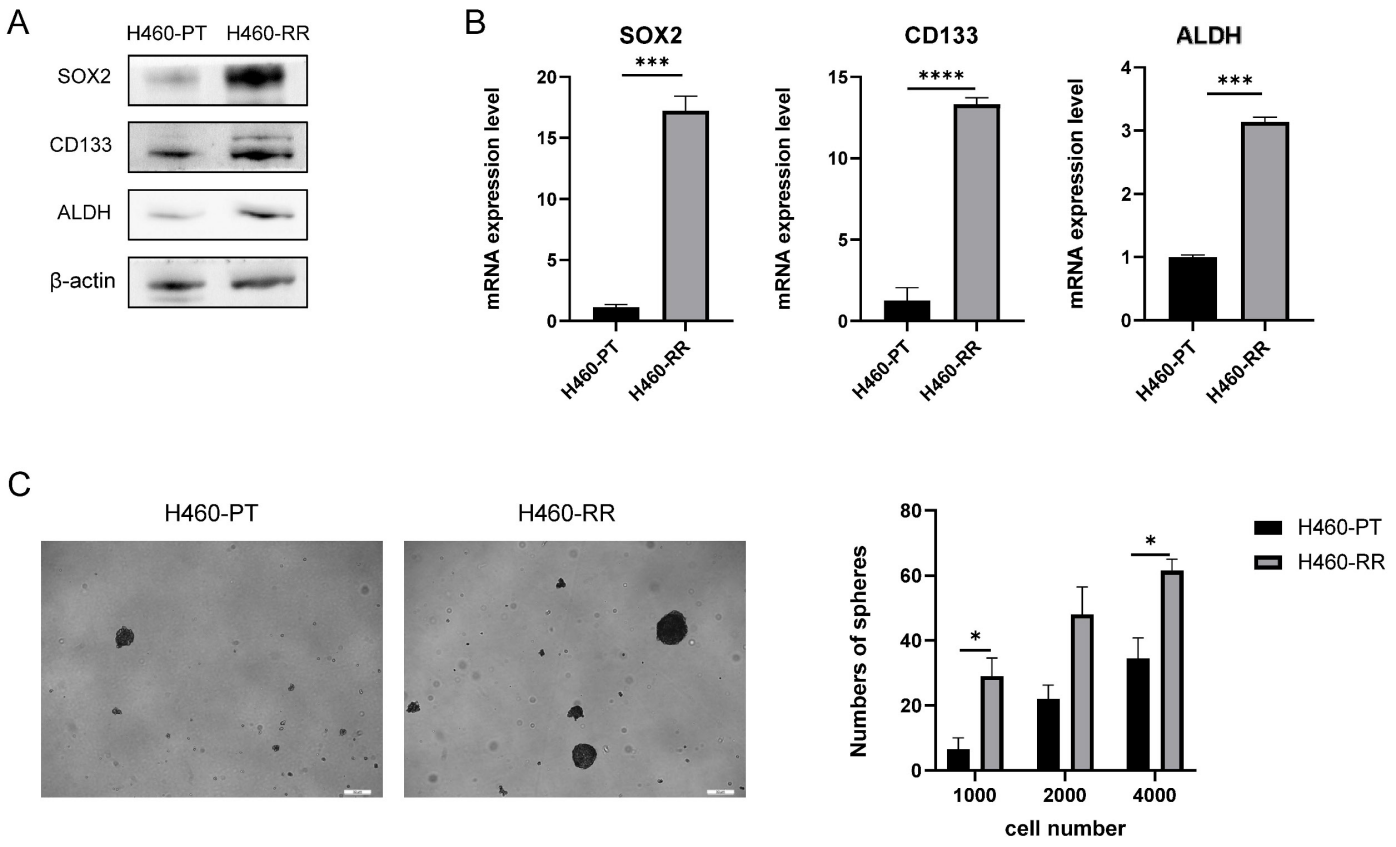
** CSC phenotype of radioresistant cells was enhanced.** (**A**) CD133, ALDH, and SOX2 protein expression levels in H460-PT and H460-RR were detected by western blot. (**B**) qRT-PCR to detect the expression levels of CSC markers CD133, ALDH, SOX2 mRNA in H460-PT and H460-RR. (**C**) Sphere-forming assay to detect the number of sphere-forming cells in H460-PT and H460-RR at the cell inoculation numbers of 1000, 2000, and 4000, respectively.

To compare the differences in stemness phenotypes between H460 parent cells and radioresistant cells, we examined the expression of common CSC markers CD133, ALDH, and SOX2 at the protein and mRNA level by western blot and qRT-PCR respectively. The western blot results revealed that compared with H460-PT, the protein expression of CD133, ALDH and SOX2 were all significantly increased in H460-RR (**Figure 3A**). Meanwhile, qRT-PCR results suggested that CD133, ALDH, and SOX2 mRNA expressions were significantly increased in H460-RR with statistically significant differences (p < 0.05, **Figure 3B**).

It was found that the growth as multicellular three-dimensional spheres in the low-adherent environment is an important characteristic of the stemness and self-renewal ability of tumor cells, and the sphere-forming assay is commonly used to identify cell stemness *in vitro* 33. Therefore, we used sphere-forming assays to analyze the stemness characteristics of H460 parental cells and radioresistant cells. H460-PT and H460-RR were inoculated in 24-well ultra-low adhesion plates in the numbers 1000, 2000, and 4000 in turn, and the cell sphere formation status was observed after 7 days of culture as shown in Figure 2C left. And the results suggested that the number of spheres in H460-RR was more than that in H460-PT (p<0.05, **Figure 3C**). All the results demonstrated that the radioresistant cells showed a trend of dedifferentiation and enhanced expression of stemness characteristics.

### SOX2 overexpression promotes the migration capacity of NSCLC cells

**Figure 4:**
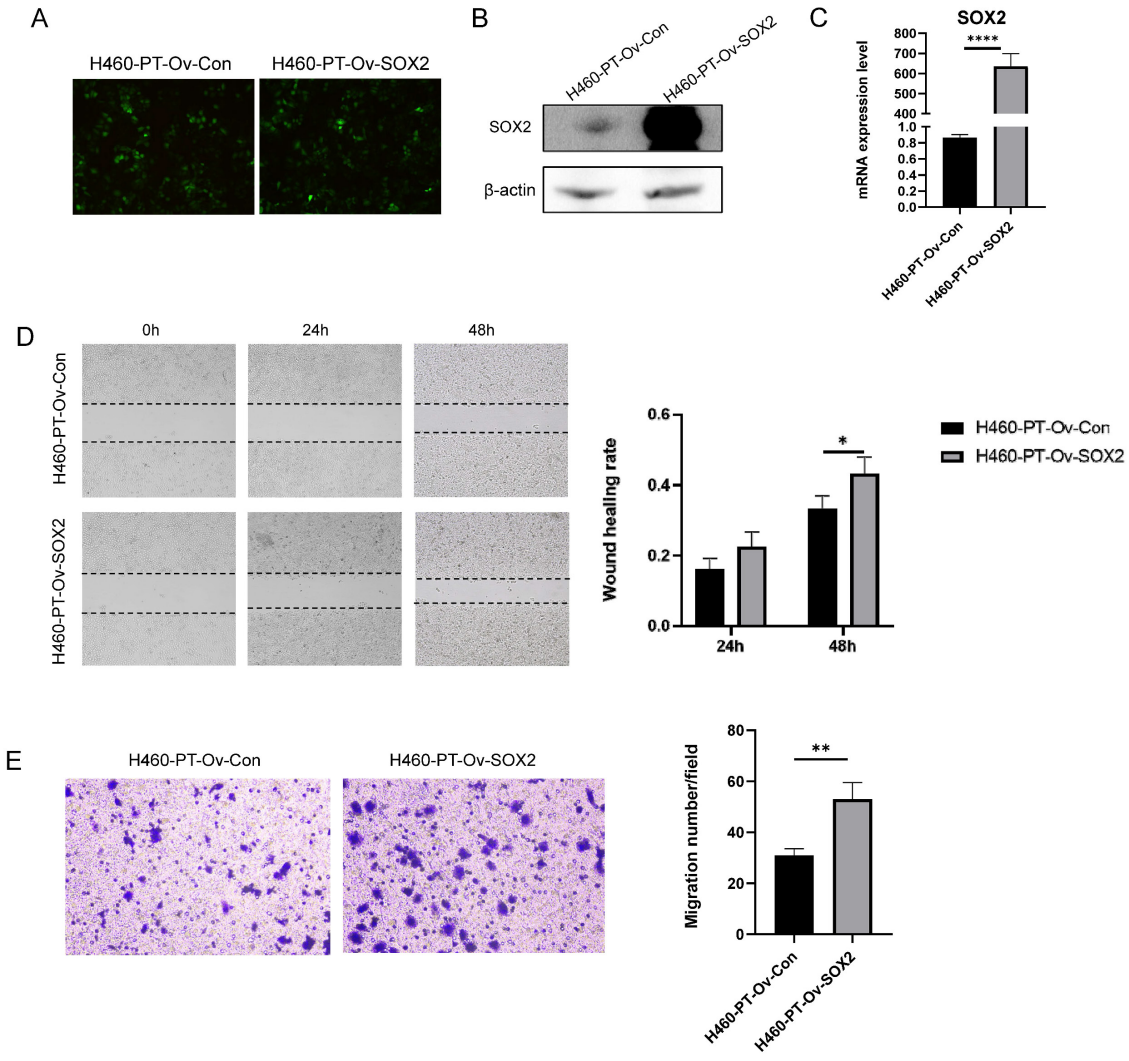
** SOX2 promotes the migration capacity of NSCLC cells.** (**A**) Fluorescence intensity of H460-PT at 48h after lentiviral transfection. (**B**) Western blot detection of intracellular SOX2 protein expression level after lentivirus transfection. (**C**) qRT-PCR detection of SOX2 mRNA expression level in lentivirus-transfected cells. (**D**) wound healing assay to detect the ability of cell migration after up-regulation of SOX2 expression. (**E**) Transwell assay to detect the migratory motility of cells after up-regulation of SOX2 expression.

The previous results showed a significant enhancement of SOX2 expression at both protein and mRNA levels in H460-RR induced by multiple radiotherapies, suggesting that SOX2 may play an important role in the regulation of NSCLC radiosensitivity. To further investigate the roles of SOX2 in NSCLC, we transfected H460-PT with a fluorescent lentiviral vector to upregulate SOX2 expression and observed the transfection efficiency using fluorescence microscopy after 48 h. The cells were in good condition (**Figure 4A**). The efficiency of overexpression was further verified by western blot and qRT-PCR, and the results showed that SOX2 was significantly increased in both protein and mRNA levels in the overexpression group compared to the control group (p<0.05, **Figure 4B, 4C**), demonstrating that the transfection was successful and ready for subsequent experiments.

To investigate whether SOX2 is capable of facilitating invasion and migration in NSCLC, we performed wound healing experiments on transfection cells and calculated the healing efficiency. The migration distances of cells in each group at 24 and 48 h after scratching are shown below (**Figure 4D** left). By calculating the percentage of scratch healing, it was found that the migration ability of cells in the SOX2 overexpression group was significantly higher than that of the control group (p<0.05, **Figure 4D** right). Similarly, Transwell assay results suggested that upregulation of SOX2 expression enhanced H460-PT migration ability (p<0.05, **Figure 4E**).

### SOX2 down-regulation suppresses the migration capacity of NSCLC cells

**Figure 5:**
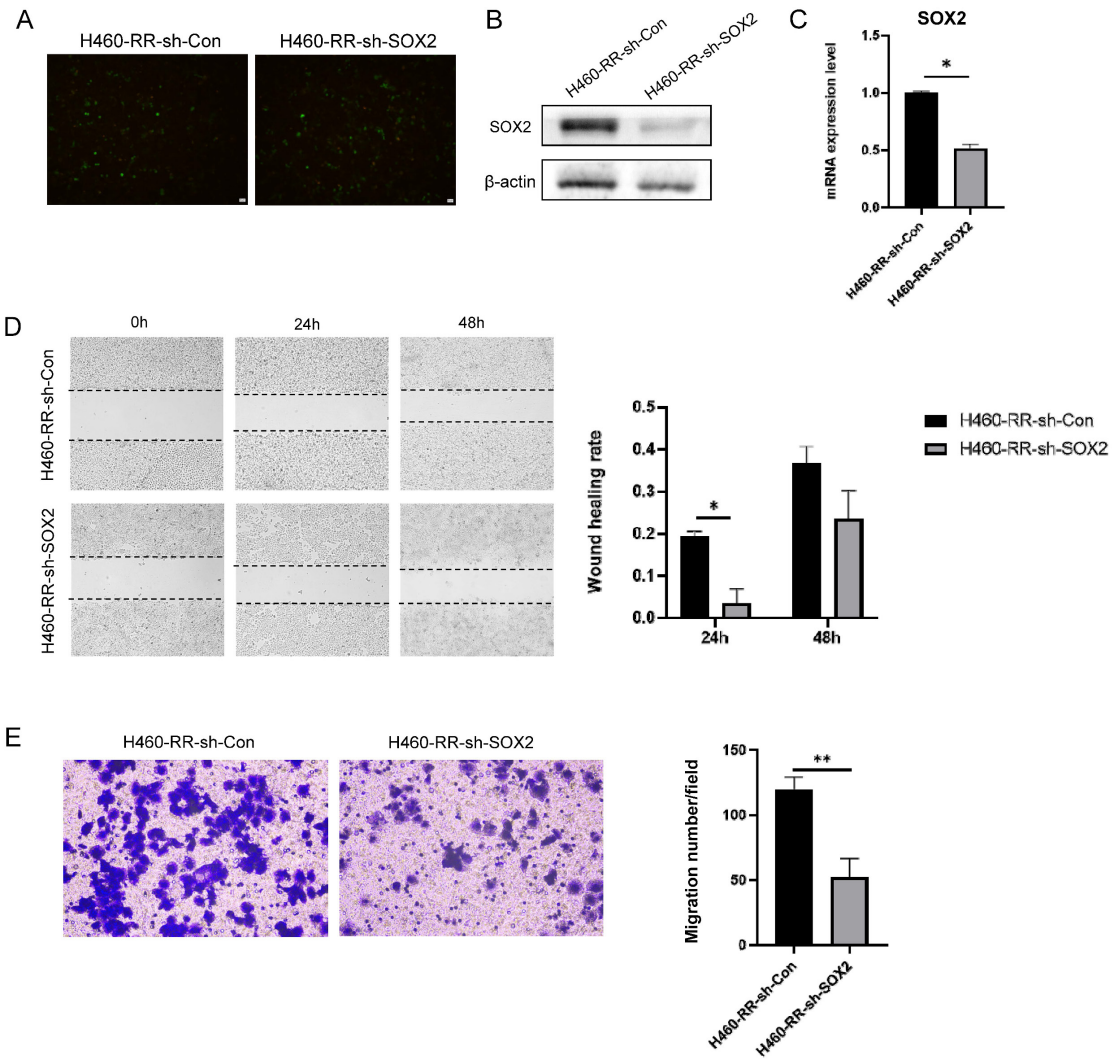
** SOX2 knockdown suppresses the migration capacity of NSCLC cells.** (**A**) Fluorescence expression of H460-RR at 48h after lentiviral transfection. (**B**) Western blot detection of SOX2 protein expression level in H460-RR after lentivirus transfection. (**C**) qRT-PCR assay of SOX2 mRNA expression level in lentivirus transfected cells. (**D**) wound healing assay to detect the ability of cell migration after down-regulation of SOX2 expression. (**E**) Transwell assay to detect the migratory motility of cells after down-regulation of SOX2 expression.

To verify the effect of SOX2 on the migratory motility of cells, SOX2 knockdown was performed on radioresistant cells H460-RR using a fluorescently labeled lentiviral vector. The intracellular fluorescent expression at 48h after lentiviral transfection is shown in **Figure 5A**. Whether SOX2 was stably knocked down in H460-RR was verified by western blot and qRT-PCR. The results showed that SOX2 was significantly decreased at both protein and mRNA levels in cells transfected with lentivirus compared with control cells transfected with null (**Figure 5B, 5C**).

Similarly, wound healing experiments were performed on transfection cells and the migration distances of cells in each group at 24 and 48 h after scratching are shown below (**Figure 5D** left). The migration ability of cells in the SOX2 silencing group was significantly lower than that of the control group (p<0.05, **Figure 5D** right). Transwell assay results suggested that downregulation of SOX2 attenuated H460-RR migration motility (p<0.05, **Figure 5E**). Therefore, SOX2 can regulate the migration capacity of NSCLC cells.

### SOX2 overexpression enhanced the resistance of H460 cells to irradiation

**Figure 6:**
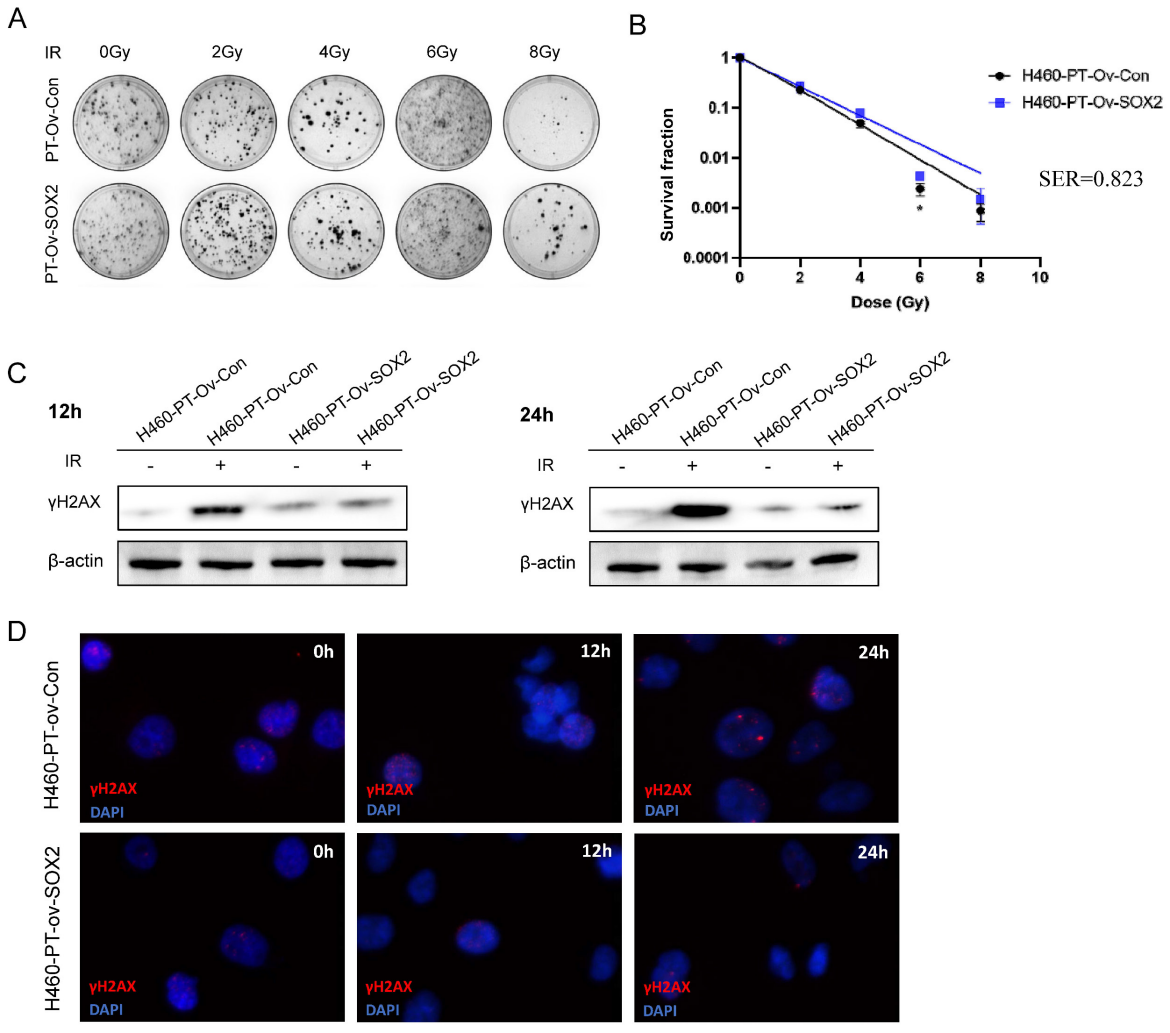
** SOX2 overexpression enhanced the resistance of H460 cells to irradiation.** (**A**) Clone formation assay to detect the number of clone formation in two groups of cells under different irradiation doses. (**B**) One-click multi-target model to fit the cell survival curve. (**C**) Western blot to detect the expression level of γH2AX protein after cellular radiotherapy. (**D**) Immunofluorescence assay to detect γH2AX fluorescence intensity after cellular radiotherapy. H460-PT-Ov-Con: H460 parental cells were transduced with empty vector, H460-PT-Ov-SOX2: H460 parental cells were transduced with the vector containing SOX2.

Given our previous finding that the expression of SOX2 was significantly higher in the radioresistant cells and was related to enhanced migration ability, we hypothesized that SOX2 may be involved in regulating cellular dedifferentiation, leading to radiotherapy resistance. To further investigate whether SOX2 upregulation affects the radiotherapy resistance of NSCLC cells, we verified the radiosensitivity between the control group H460-PT-Ov-Con and the overexpression group H460-PT-Ov-SOX2 through colony formation assay. And the results showed that compared with H460-PT-Ov-Con, the number of cell clone in H460-PT-Ov-SOX2 was increased (**Figure 6A**), and the cell survival curves suggested that the cell survival fraction were higher in the SOX2 overexpression group than in the control group (**Figure 6B**), suggesting that SOX2 overexpression enhanced the cellular tolerance to radiotherapy.

H460-PT-Ov-Con and H460-PT-Ov-SOX2 were exposed to 6 Gy of radiation, and the γH2AX protein expression of the two groups of cells was detected by western blot at different time points after radiotherapy. The results showed that at 12 and 24 h after irradiation, the γH2AX expression of H460-PT-Ov-SOX2 was significantly lower than that of H460-PT-Ov-Con (**Figure 6C**). The immunofluorescence experiment showed that the fluorescence intensity of γH2AX in the SOX2 overexpression group was weaker than that in the control group after irradiation, and it was most obvious at 24 h after irradiation (**Figure 6D**), which verified that the upregulation of SOX2 expression could promote DNA damage repair ability in H460 cells. All the results suggested that upregulation of SOX2 expression can enhance the resistance of NSCLC cells to radiation.

### SOX2 down-regulation attenuated the radiation resistance of H460-RR

**Figure 7:**
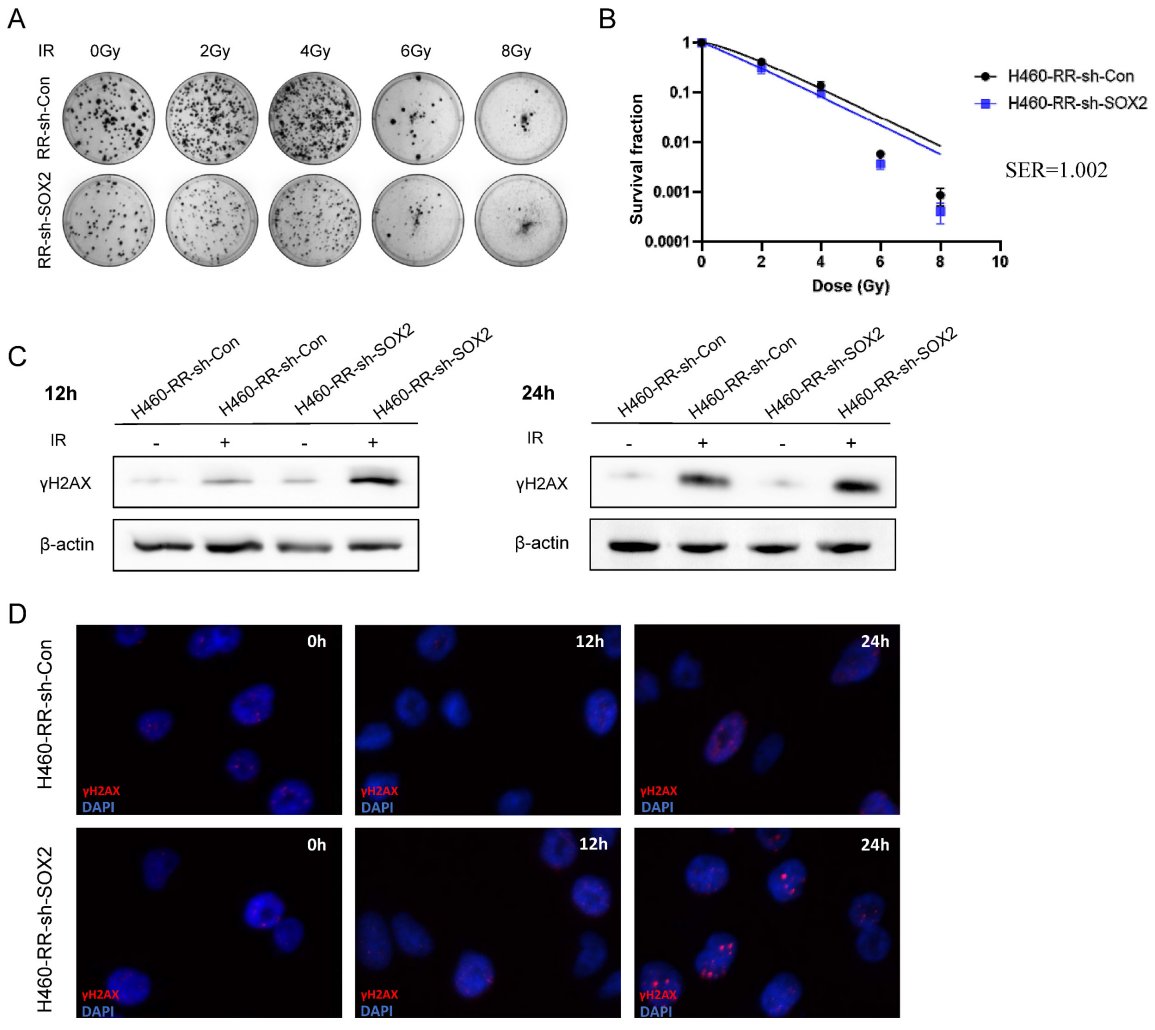
** SOX2 down-regulation improves sensitivity to irradiation in H460-RR.** (**A**) Clone formation assay to detect the ability of cell clone formation after radiotherapy. (**B**) One-click multi-target model to fit the cell survival curve. (**C**) Western blot to detect γH2AX protein expression levels at 12 and 24 h after radiotherapy. (**D**) Immunofluorescence assay to detect the fluorescence intensity of γH2AX at 12 and 24 h after radiotherapy. H460-RR-sh-Con: H460-RR cells were transduced with empty vector, H460-RR-sh-SOX2: silencing of SOX2 in H460-RR cells.

We have found that H460 cells overexpressing SOX2 have reduced sensitivity to radiotherapy and appear to have a radioresistance profile. To further explore whether SOX2 plays a regulatory role in radiotherapy resistance in NSCLC, Colony formation assay was performed to detect the radiosensitivity after down-regulated SOX2 expression. The results showed that the number of clone formation in SOX2 knockdown group cells was less than that in control group cells (**Figure 7A**), and the survival fraction after each dose of irradiation was lower than that of the control group (**Figure 7B**). It is suggested that there is a trend of decreased radiation tolerance of H460-RR after SOX2 expression down-regulation.

The western blot and immunofluorescence assays examined the effect of SOX2 expression downregulation on DNA repairability. The western blot results showed that the expression of γH2AX protein was significantly higher in the SOX2-silenced group than in the control group at 12 and 24 h after 6Gy irradiation (**Figure 7C**). Immunofluorescence results showed that the γH2AX fluorescence intensity was not significantly different between the two groups at 12 h post-irradiation. However, at 24 h post-irradiation, the γH2AX fluorescence was higher in the SOX2 knockdown group than in the control group (**Figure 7D**), indicating that the down-regulation of SOX2 expression diminished the DNA damage repair ability of radiotherapy-resistant cells. It can be concluded that the down-regulation of SOX2 expression level can reduce the radiotherapy resistance of NSCLC cells to some extent.

### SOX2 promotes radiation resistance via regulating dedifferentation of NSCLC cells

Up-regulation of SOX2 expression induced radiotherapy resistance in H460 cells, while down-regulation of SOX2 expression attenuated radiation tolerance and enhanced radiotherapy sensitivity in H460 cells, suggesting that SOX2 may be an important regulator of the development of radiotherapy resistance in NSCLC cells. It has been demonstrated that the presence of CSC is a key factor contributing to radiotherapy resistance 22. SOX2, as an important transcription factor involved in maintaining cell stemness regulating cell differentiation 28, may influence cellular radiotherapy resistance by regulating stemness expression.

**Figure 8:**
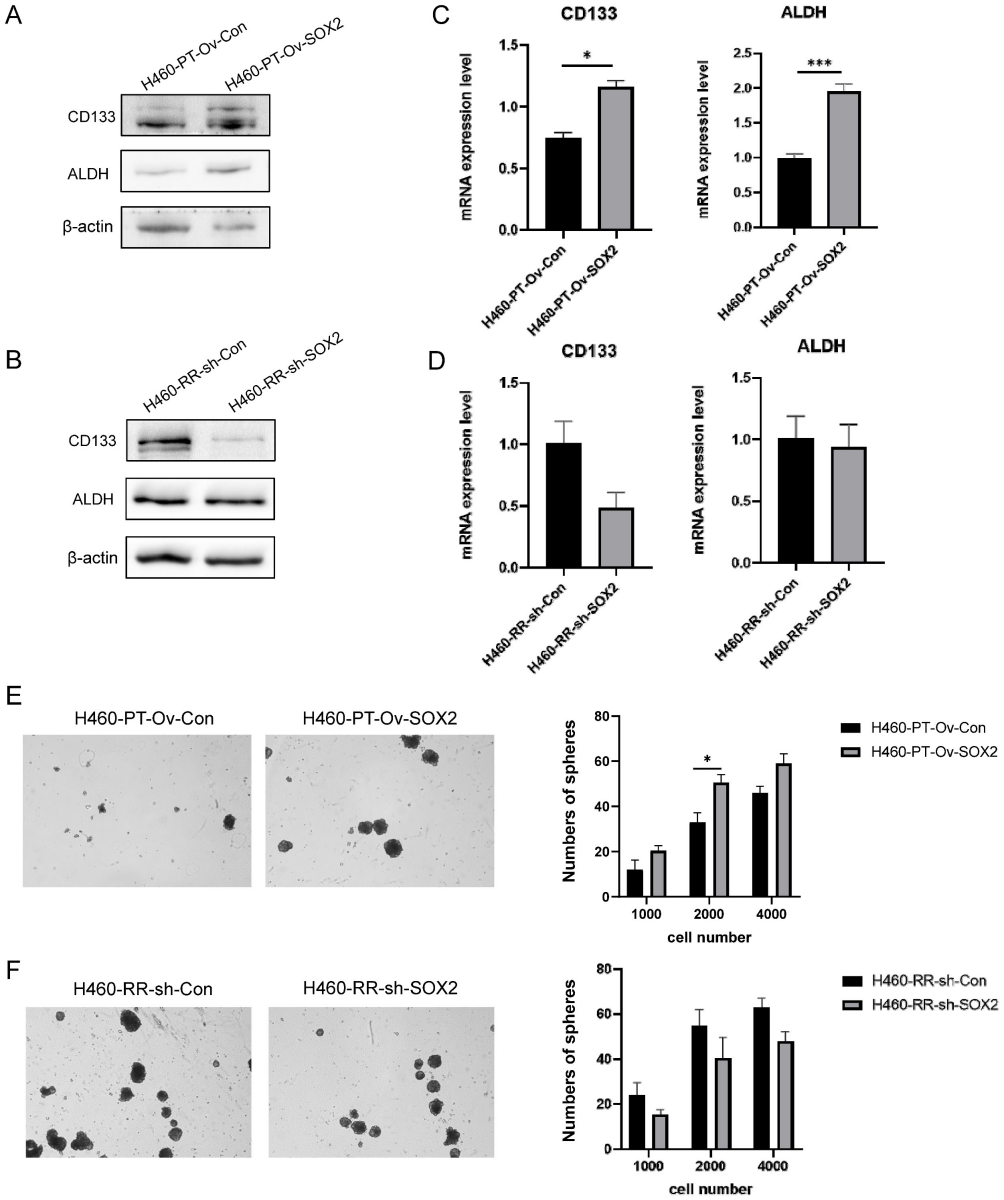
** SOX2 promotes radiation resistance via regulating dedifferentation of NSCLC cells.** (**A**) and (**B**) western blot detection of CD133 and ALDH protein expression in H460 cells at different expression levels of SOX2. (**C**) and (**D**) qRT-PCR to detect CD133 and ALDH mRNA expression in H460 cells at different expression levels of SOX2. (**E**) and (**F**) Sphere-forming assay to detect the sphere-forming ability of cells after up-and down-regulation of SOX2 expression.

We examined the stemness expression changes of H460-PT and H460-RR cells after lentiviral transfection by various experimental methods. western blot results showed that with the upregulation of SOX2 expression in H460-PT, the protein expression levels of CD133 and ALDH appeared significantly increased (**Figure 8A**) while silencing of SOX2 in H460-RR resulted in CD133 expression decreased and ALDH did not show significant changes (**Figure 8B**). The changes in CD133 and ALDH mRNA expression levels in H460 cells after viral transfection were detected by qRT-PCR, and the results showed that the expression of CD133 and ALDH in H460-PT increased with SOX2 overexpression (**Figure 8C**), while downregulation of SOX2 expression in H460-RR attenuated CD133 expression (**Figure 8D**), which was consistent with the protein level changes was largely consistent. The effect of SOX2 changes on cell stemness characteristics was further analyzed by a sphere-forming assay. The results showed that the upregulation of SOX2 expression increased the number of sphere-forming cells and significantly improved the sphere-forming ability (**Figure 8E**), while the number of sphere-forming cells showed a decreasing trend with the silencing of SOX2 expression (**Figure 8F**). These results suggest that SOX2 can promote cell dedifferentiation and enhance the cell stemness phenotype of NSCLC, leading to radiotherapy resistance.

### High expression of SOX2 predicts tumor progression and poor prognosis in NSCLC

**Figure 9:**
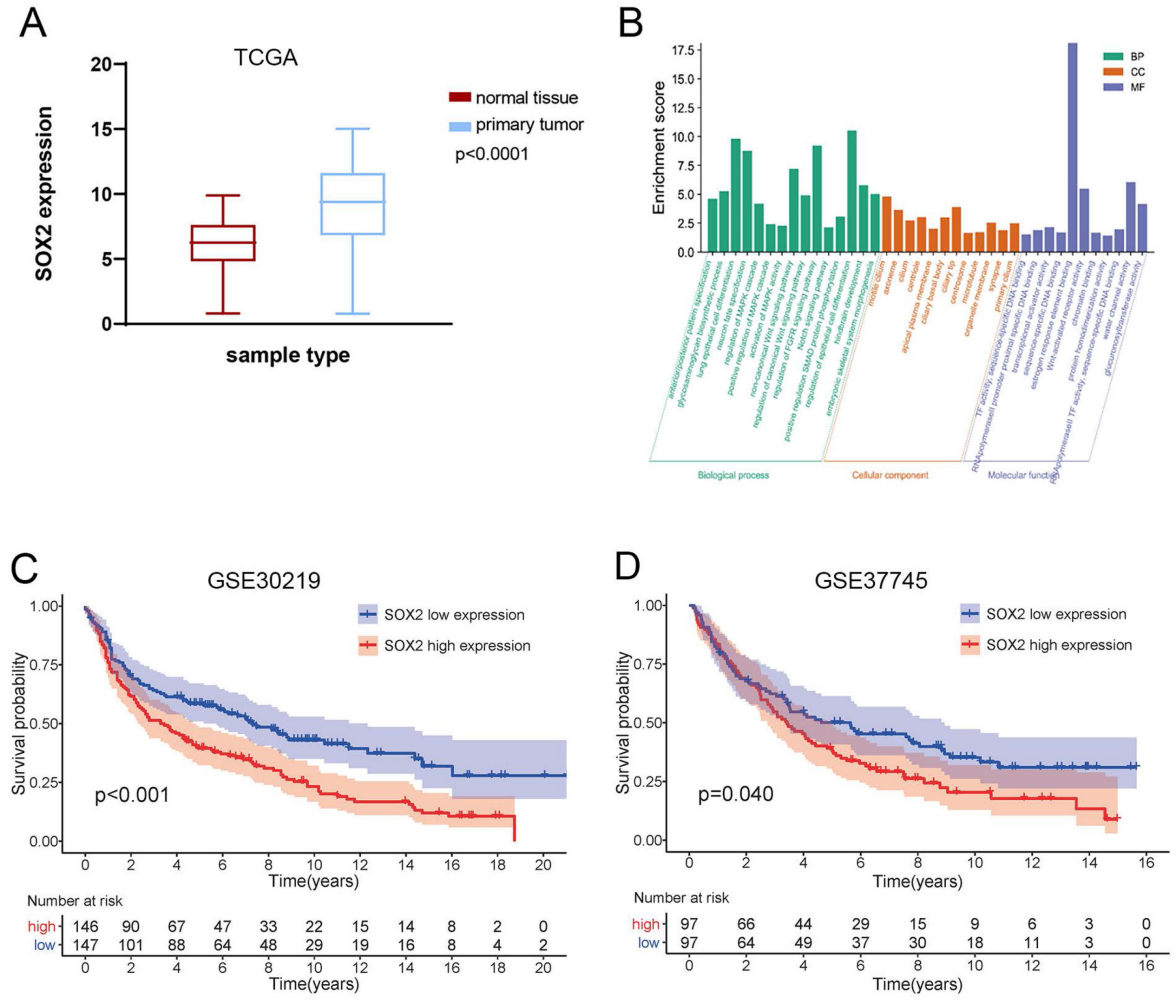
** Expression and prognosis value of SOX2 and function analysis.** (**A**) TCGA database analysis of SOX2 expression differences in lung cancer and paracancerous tissues. (**B**) Kaplan-Meier survival curve comparing NSCLC patients with high and low expression of SOX2 in GSE30219. (**C**) Kaplan-Meier survival curve comparing NSCLC patients in GSE37745. (**D**) GO enrichment analysis of SOX2-related biological functions.

The expression of SOX2 was significantly higher in tumor tissues than in adjacent normal tissues of NSCLC samples in TCGA (**Figure 9A**). Kaplan-Meier survival analysis showed that patients with high SOX2 expression had shorter survival than those with low SOX2 expression in the GSE30219 (**Figure 9B**) and GSE37745 (**Figure 9C**). The gene enrichment analysis showed that SOX2 was associated with multiple cellular components, biological processes, and molecular functions (**Figure 9D**), such as the WNT, Notch, and MAPK signaling pathways. It is suggested that SOX2 plays an important role in radiotherapy-induced CSC phenotypes.

## Discussion

Radiotherapy is a common and effective treatment modality for NSCLC, mainly through ionizing radiation causing irreparable DNA double-strand breaks thereby killing tumor cells, reducing tumor load, and improving local control rate 3. However, some patients still experience recurrent disease or tumor cell metastasis after radiotherapy, resulting in a five-year overall survival rate of patients still below 15%, which is mainly due to the occurrence of resistance to radiotherapy 4. Therefore, it is important to explore the relevant factors involved in regulating the sensitivity to radiotherapy in NSCLC and their possible mechanisms to overcome the resistance to radiotherapy and develop more effective treatment strategies.

An increasing number of studies have pointed out the significance of the presence of CSC in radiotherapy resistance and tumor recurrence after radiotherapy^22^.CSC can improve their resistance to radiation through mechanisms such as regulation of the cell cycle, rapid repair of DNA damage, reduction of apoptosis, resistance to cell damage caused by oxidative reactions, and interaction with the tumor microenvironment^23^, ^38^, ^39^. Therefore, even though radiotherapy can kill most of the tumor cells, CSC survives and reassemble due to their resistance to radiotherapy, which eventually leads to radiotherapy failure and tumor recurrence and metastasis. In recent years, it has been found that radiotherapy can cause therapeutic enrichment of CSC. In gliomas, a higher proportion of CD133^+^ tumor cells survived radiotherapy than CD133^-^ cells and showed post-radiotherapy enrichment, and these CD133^+^ cells are also thought to be the source of tumor recurrence after radiotherapy 16. In prostate cancer, surviving cells after high-dose radiation have an enhanced sphere-forming capacity and high expression of the CSC marker CD44, and this stemness profile is progressively enhanced within one week after irradiation 40. In breast cancer, the CD44^+^/CD24^-^ stem cell population was significantly increased in cancer cells induced by radiotherapy 41. Similarly, in NSCLC, the percentage of CD133^+^ cells increased significantly after radiotherapy, with a concomitant increase in cell sphere-forming capacity 42. In our study, H460 cells screened by multiple radiotherapy induction showed significant resistance to radiotherapy, i.e., H460-RR, and the expression of CD133, ALDH, and SOX2, which represent the CSC phenotype, were significantly upregulated in these cells compared to the parental cells, and the sphere-forming ability was also significantly enhanced, consistent with the above findings.

Dysregulated SOX2 expression plays an important role in the development of several epithelial cell carcinomas and is associated with malignant tumor progression and poor prognosis 30, 43. We observed differential expression of SOX2 in lung cancer and paraneoplastic tissues and the WNT, Notch, and MAPK signaling pathways associated with SOX2 are usually aberrantly activated in CSC and play an important role in radiotherapy-induced CSC phenotypes 24. In addition, it was found that in cervical cancer, SOX2 can modulate cellular radioresistance by altering cell proliferation, apoptosis, and cell cycle changes after radiation through the Hedgehog signaling pathway 36. In colorectal cancer, radiotherapy activates the PI3K/AKT signaling pathway, which in turn causes upregulation of SOX2 expression, ultimately leading to increased CD44^+^ cells and enhanced radioresistance^44^. This shows that SOX2 is a key factor mediating radiotherapy resistance. In the present study, the high expression of SOX2 in radiotherapy-resistant cells has been verified, and we further found that after altering the expression level of SOX2 using lentiviral transfection, the radiation tolerance of NSCLC cells was enhanced when SOX2 was overexpressed, and conversely, when SOX2 expression was downregulated, the cells showed a decreasing trend of radioresistance, indicating that SOX2 has a regulatory role.

SOX2, as a stem cell marker, is involved in cell stemness regulation to maintain cell pluripotency 27. In breast cancer, the tumorigenicity of tumor cells can be reduced by suppressing SOX2 expression, and restoration of SOX2 expression facilitates CSC growth, and similar phenomena have been verified in nasopharyngeal carcinoma 45, 46. In colon cancer, tumor cells with high SOX2 expression have an enhanced sphere-forming ability and are accompanied by elevated expression of the CSC markers CD44 and CD24^47^. In pancreatic cancer, overexpression of SOX2 induced cell proliferation and spherogenesis, promoted cancer cell dedifferentiation, and increased intracellular CD44, and ALDH expression levels 48. In our study, the same association was found between SOX2 and CSC phenotype: upregulation of SOX2 caused elevated expression of NSCLC CSC markers CD133 and ALDH and enhanced cell sphericity, while downregulation of SOX2 decreased cellular CD133 expression and sphericity, and changes in cell stemness characteristics corresponded to altered sensitivity to radiotherapy. Here we noted that silencing SOX2 had no significant effect on ALDH, suggesting that the regulation of SOX2 on NSCLC cell stemness may be more inclined to the effect on CD133 expression, while the expression level of ALDH may also be regulated by other factors 49.

The efficacy of radiotherapy depends on the extent of DNA damage in tumor cells, and radiation can cause sublethal damage in addition to lethal damage, which gradually accumulates and eventually leads to cell death, so the DNA damage repair capacity of cells is a key factor in determining the sensitivity of cells to radiotherapy 50. In contrast, CSC exhibit a higher DNA repair capacity than ordinary tumor cells. γH2AX is a sensor of the DNA damage response and appears rapidly after radiation-induced DNA damage, helping to identify the damage site and promoting signaling and activation of cell cycle checkpoint proteins 37. Previous studies found that γH2AX expression was significantly lower in CSC than in non-CSC in breast and pancreatic cancers after radiotherapy 51, 52. In gliomas, γH2AX expression was similar in CD133^+^ and CD133^-^ cells after radiotherapy, but the faster decrease in γH2AX in CD133^+^ cells suggested more efficient damage repair 16. Another study observed that upregulation of SOX2 expression in glioma stem cells was able to reduce the expression level of γH2AX after radiotherapy, which by further analysis was found to be possibly related to the involvement of SOX2 in the Homologous Recombination (HR) repair pathway 53. Our study showed that within 24 h after radiotherapy, cells in the SOX2 overexpression group had lower levels of γH2AX expression and also recovered more quickly to unirradiated levels, whereas downregulation of SOX2 resulted in significantly higher levels of γH2AX expression and correspondingly slower recovery after irradiation, suggesting that SOX2 expression levels can affect the ability to repair DNA damage, which is consistent with altered SOX2 expression in cells with radiotherapy sensitivity and stemness alterations were consistent with the altered expression of SOX2.

The ability of tumor cells to migrate and invade is a major contributor to radiotherapy resistance and post-treatment recurrence and metastasis 54. It has been demonstrated that radiotherapy-resistant cells have greater viability and motility 34. Similarly, it was observed in our experiments that radiotherapy-resistant cells induced by multiple radiation treatments exhibited enhanced migratory motility. It has been shown that SOX2 enhances the migration and invasion of cancer cells and promotes tumor metastasis 55, 56. The migratory invasion ability of cells is known to be regulated by E-cadherin, N-cadherin, Vimentin, and the transcription factors Snail, Slug, etc., and inhibition of E-cadherin, Vimentin or enhanced expression of N-cadherin, Snail can reduce intercellular adhesion and thus promote cell invasion and metastasis 57. In colon cancer, enhanced SOX2 expression reversed the downregulation of CD133 and CD44 and the upregulation of E-cadherin 58, and inhibition of SOX2 decreased Snail expression, leading to a decrease in the invasive migratory capacity of cells 56. In pancreatic cancer, overexpression of SOX2 was able to downregulate E-cadherin and upregulate Snail, which enhanced cell invasive migration 48. In cervical cancer, compared to SOX2-negative cells, tumor cells that endogenously express SOX2 exhibit an enhanced stemness phenotype along with decreased expression of E-cadherin and Vimentin and a high degree of invasive metastatic capacity 59. Our experimental results showed that upregulation of SOX2 expression enhanced the migration ability of NSCLC cells and silencing of SOX2 caused diminished cell migration ability, which combined with changes in cellular resistance to radiotherapy, suggested that SOX2 might affect radiotherapy sensitivity by regulating cell migration movement, but the specific regulatory pathway of SOX2 on migration invasion of NSCLC cells needs to be investigated in depth.

## Conclusions

In this study, we constructed the NSCLC radioresistance cell line induced by multiple radiotherapy treatments and confirmed that they could develop radioresistance. And SOX2 promotes cellular resistance to radiotherapy by regulating NSCLC cell dedifferentiation and enhancing their DNA damage repair ability.

## Abbreviations

NSCLC: Non-small cell lung cancer
CSC: Cancer stem cell
RT: Radiotherapy
RR: Radioresistance
SOX2: SRY-box transcription factor 2
ALDH: Aldehyde dehydrogenase
